# Synthesis and Optimization of Chitosan Ceramic-Supported Membranes in Pervaporation Ethanol Dehydration

**DOI:** 10.3390/membranes8040119

**Published:** 2018-11-30

**Authors:** Mahdi Nikbakht Fini, Sepideh Soroush, Mohammad Mehdi Montazer-Rahmati

**Affiliations:** 1 Section of Chemical Engineering, Department of Chemistry and Bioscience, Aalborg University, 6700 Esbjerg, Denmark; 2 Research Center for Membrane Separation Processes, Faculty of Chemical Engineering, Iran University of Science and Technology, Tehran 1684613114, Iran; s.soroush7@gmail.com; 3 School of Chemical Engineering, College of Engineering, University of Tehran, Tehran 1417614418, Iran; mrahmati@ut.ac.ir

**Keywords:** chitosan, hybrid membrane, ceramic supports, pervaporation, ethanol dehydration

## Abstract

In the present work, ceramic-supported chitosan hybrid membranes were prepared for the pervaporation dehydration of ethanol. Mullite and combined mullite-alumina (50% alumina content) tubular low-cost ceramic supports were fabricated, and their influence on membrane performance was compared to a commercial α-alumina support. The membrane preparation parameters were different ceramic supports and the concentration of chitosan solution (varying from 2 wt.% to 4 wt.%). The supports and hybrid membranes were characterized by field emission scanning electron microscopy (FE-SEM) and contact angle measurements. The results show, with increasing chitosan concentration, the permeability decreases, and selectivity increases. It was also found that the separation factor decreases with increasing feed temperature and feed water content, while the permeation flux increases. The membrane that was coated on α-alumina support with a 3 wt.% chitosan concentration exhibited the best pervaporation performance, leading to a permeation flux and separation factor of 352 g·m^−2^·h^−1^ and 200 for 90 wt.% ethanol in feed at 60 °C, respectively.

## 1. Introduction

Dehydration of alcohols, particularly ethanol, due to the formation of an azeotrope is one of the applications of the pervaporation process. Pervaporation in comparison with azeotropic distillation or extractive distillation consumes less energy and does not require a third component [1,2,3,4,5]. In recent decades, dehydration of ethanol by polymeric, ceramic, and zeolite membranes has received increasing attention [6]. Although ceramic membranes have more chemical, physical and thermal stability compared to polymeric membranes, the high costs and difficulty of fabrication limit their application in the industry [7]. Various hydrophilic polymers such as PVA, alginate, chitosan, and polyamides have been used in pervaporation of ethanol [6,8,9,10]. Among these polymers, chitosan has attracted much attention in this field because of its relatively low price, appropriate film-forming, non-toxicity, facile structure modification and high hydrophilicity [11,12,13].

There are some restrictions on the use of polymeric membranes such as low mechanical and thermal stability, loss of surface integrity and poor processability. In addition, one of the other difficulties in polymeric membranes is the stretching of polymer chains and the formation of voids in aqueous solutions, known as swelling. To overcome these problems for chitosan membranes, blending with other polymers, for example PVA [14,15,16], alginate [17,18], hydroxyethylcellulose [19,20], Nylon 66 [21], etc.; cross-linking with phosphoric acid [22], glutaraldehyde [13], APTEOS [23], GPTMS [24], etc.; or incorporating inorganic particles for instance zeolites [12,25], carbon nanotubes [26], SiO_2_ [27], TiO_2_ [28], etc., have been proposed by researchers. Using a porous ceramic substrate as support for selective polymers can be effective for achieving defect-free surface and high mechanical and thermal resistance. In these organic-inorganic membranes, the polymer-coated dense layer is responsible for separation and the porous ceramic support provides mechanical, thermal, and chemical stability and improves the permeability and processability [29,30,31].

Despite the fact that numerous ceramic supports such as α-alumina [32], zirconia [31], γ-alumina [30], Silica [33], kaolin [34], etc. have strongly influenced the performance of polymeric membranes, their high cost makes them less attractive for industrial applications. Research must continue in this field to make membranes more competitive. A number of various inexpensive raw materials, such as natural clay, apatite powder, dolomite, kaolin, bauxite, and mineral coal fly ash have been used for production of ceramic membranes that could be also employed as ceramic supports for polymeric selective layer [35]. Therefore, it was decided to prepare an inexpensive support using kaolin as a raw material. The extrusion method was applied to fabricate tubular ceramic supports providing a higher effective area in a lower space.

In this study, mullite and combined mullite-alumina tubular supports were prepared. The chitosan selective layer was coated on the outer surface of the supports by the simple dip-coating technique. The objective of this work was to compare the effect of the prepared mullite and mullite-alumina low-cost ceramic supports with commercial α-alumina supports in the pervaporation process. In addition to the support type, the influence of chitosan concentration on membrane performance was investigated at three levels. The performance of the synthesized membranes was assessed by the pervaporation dehydration of ethanol and their characterization was achieved using both FE-SEM and the contact angle measurement. The effect of operational conditions on the pervaporative ethanol dehydration was also examined by using different feed concentrations as well as feed temperatures.

## 2. Materials and Methods

### 2.1. Materials

Chitosan (medium molecular weight) was obtained from Aldrich (St. Louis, MO, USA). Acetic acid and absolute ethanol were of reagent grade and manufactured by Merck (Darmstadt, Germany). Deionized water was produced in the laboratory by ELGASTAT (London, UK). Kaolin powder, which used in the preparation of supports, was purchased from the Iran China Clay Industries Company (Tabriz, Iran). An aluminum oxide with a purity of 99.6 wt.%, was supplied by the Fibrona Company (Tehran, Iran). Chemical analysis of kaolin and aluminum oxide are presented in Table 1 and Table 2. α-alumina support with a pore size of 600 nm was obtained from the Pall company (New York, NY, USA).

### 2.2. Ceramic Support Preparation

In this work, different ceramic supports, mullite, mullite-alumina, and α-alumina were used. Mullite and mullite-alumina tubular supports were fabricated from kaolin clay and Al_2_O_3_ powder. As presented in our earlier work [36], tubular mullite supports (id: 10 mm, od: 13 mm, and L = 10 cm) were made by the extrusion of the homogeneous mud composed of about 62–65% kaolin and 35–38% deionized water. The wet extrudate supports were dried at an ambient temperature for 48 h and then calcinated for 3 h at 1250 °C in an electric Exciton furnace. Then to increase the porosity of supports, their free silica was removed in a 20 wt.% NaOH aqueous solution bath at 80 °C for 5 h. Ceramic supports were then washed with deionized water at 80 °C for 12 h to remove NaOH and a neutral pH was obtained. Combined mullite-alumina supports with a 50 wt.% alumina powder content were synthesized in a similar way but were calcinated at 1300 °C.

### 2.3. Membrane Preparation

To prepare the polymeric coating layer, chitosan was dissolved in a 2 wt.% acetic acid aqueous solution under stirring at 50 °C. The duration of stirring to form a homogenous solution depends on the solution concentration. Three different chitosan concentrations of 2, 3, and 4 wt.%, were used to coat the polymer on the different supports. The solution was then filtered through a mesh No. 250 to remove undissolved residue particles. The resultant solution was coated on the polished outer surface of ceramic supports by dipping them in the solution for about a minute. The coated supports were dried at room temperature for about 5 h under the slow rotation of 60 rpm and then annealed at 60 °C for 8 h in an oven to achieve complete evaporation of the solvent.

### 2.4. Membrane Characterization

The pore size of fabricated ceramic supports was determined using mercury porosimetry. The porosity of the supports was measured by the water absorption method. The morphology and structure of surface and cross-section of supports and membranes were studied by field emission scanning electron microscopy (FE-SEM) using Hitachi S4160 (Hitachi, Tokyo, Japan). The hydrophilicity of the used supports was evaluated by a contact angle measurement system made in the Nanotechnology Laboratory of the Institute of Petroleum Engineering of the University of Tehran, Tehran, Iran. In this system, the contact angle of a decane drop in contact with three different points of the ceramic supports surface was measured. The preconditioning of ceramic supports was done by polishing of outer surface of supports using various sandpapers (No. 240, 400, and 1200) while the tubular support was rotating in order to obtain a homogeneous smooth surface and no visible hole or bump was seen on the surface. The contact angle measurement error was less than 5 percent.

### 2.5. Pervaporation

**Figure 1:**
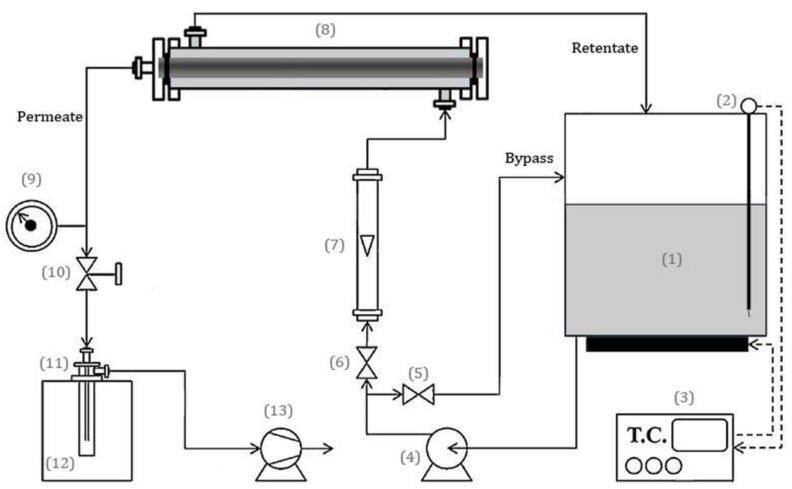
The schematic diagram of laboratory pervaporation set-up: (**1**) feed tank; (**2**) thermometer; (**3**) temperature controller; (**4**) feed pump; (**5**,**6**) Valve; (**7**) rotameter; (**8**) membrane module; (**9**) pressure gage; (**10**) vacuum regulator; (**11**) permeate collection trap; (**12**) liquid nitrogen tank; (**13**) vacuum pump.

Pervaporation dehydration of ethanol was performed using a laboratory-scale setup shown in Figure 1. The water composition in the feed mixture was 10 wt.% for all of the membranes and 10, 20, 30, 40, and 50 wt.% for the best one in order to study the feed concentration effect. The experiments were carried out at 60 °C for all of the membranes and 30, 40, 50, 60, and 70 °C for the best membrane for investigation of the temperature effect. The effective surface area of the membrane in contact with the feed was 16.33 and 11 cm^2^ for membranes coated on the fabricated mullite and mullite-alumina supports and on the commercial α-alumina support, respectively. The downstream pressure of the module was maintained at 10 Torr using a vacuum pump. The test membrane was immersed in the feed mixture for about 24 h at the process temperature to equilibrate. The permeate was collected in the liquid nitrogen cold trap. The obtained permeate was weighed on the digital laboratory balance. The permeate composition was estimated by measuring its refractive index within an accuracy of 0.00001 using an ABBE refractometer AR4 (KRUSS, Hamburg, Germany) and by comparing it to the calibration graph of refractive indices of known ethanol/water mixtures. Refractometry is a straightforward, fast, inexpensive, and accurate way of ethanol concentration determination being widely used in the food and beverage industries. Although the obtained refractive indices could be rather dependent on the operator, it is a suitable analytical method giving an accurate determination when it is used by a researcher to measure the alcohol content by comparing the value of the refractive index of a solution to that of a standard curve. All the experiments were carried out three times and the average results are reported.

The pervaporation performance of the membranes was assessed in terms of permeation flux (*J*), separation factor (α), and the pervaporation separation index (*PSI*). These were calculated using the following equations respectively:

(1) $$J=\frac{Q}{At}$$

(2) $$\alpha =\frac{Y_{w}/Y_{eth}}{X_{w}/X_{eth}}$$

(3) PSI=J×(α−1)

where *Q* is the weight of the permeate (g); *A* represents the effective area of membrane (m^2^); *t* is the experiment time (h); *Y* and *X* are the mass fractions of permeate and feed, respectively; subscripts *w* and *eth*, denote water and ethanol respectively.

## 3. Results and Discussion

### 3.1. Ceramic Supports Characterization

**Figure 2:**
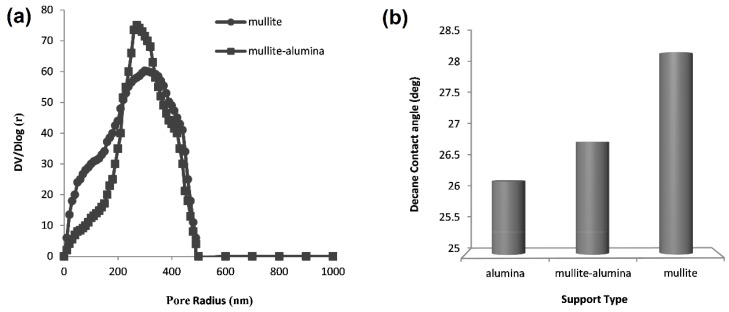
(**a**) The Mercury Porosimetry analysis of supports; (**b**) The decane drop contact angle with different ceramic supports.

Figure 2a shows the pore size distribution of the fabricated ceramic supports. As it can be seen, the average pore radius of mullite support is approximately 250 nm and that of the mullite-alumina support is 300 nm. Moreover, pore size scattering of mullite support is higher than mullite-alumina support indicating more regular and uniform distribution of mullite-alumina pores. The porosity of mullite and mullite-alumina supports was estimated by the water absorption technique as 21% and 24%, respectively. According to the manufacturer, commercial α-alumina support has an average pore radius of 300 nm and a porosity of approximately 33%.

As earlier mentioned, a contact angle measurement device was used to measure the hydrophilicity of the ceramic supports. Using this device, one can measure the contact angle of a static drop on the surface of a solid surface. Determination of the angle was done by drop shape analysis. Using the available facilities, a decane drop contact angle with the surface of mullite, mullite-alumina, and the commercial α-alumina supports can be measured. In fact, using this experiment, the affinity of the support surface for an organic drop can be determined. A smaller contact angle between the decane drop and the support surface means that the supports are more hydrophilic. According to Figure 2b, the hydrophilicity of α-alumina is more than the other two supports. Mullite-alumina stands in second place. This happens because of the intrinsic hydrophilicity of alumina particles compared to kaolin particles; the more alumina in the supports the more the hydrophilicity would be.

**Figure 3:**
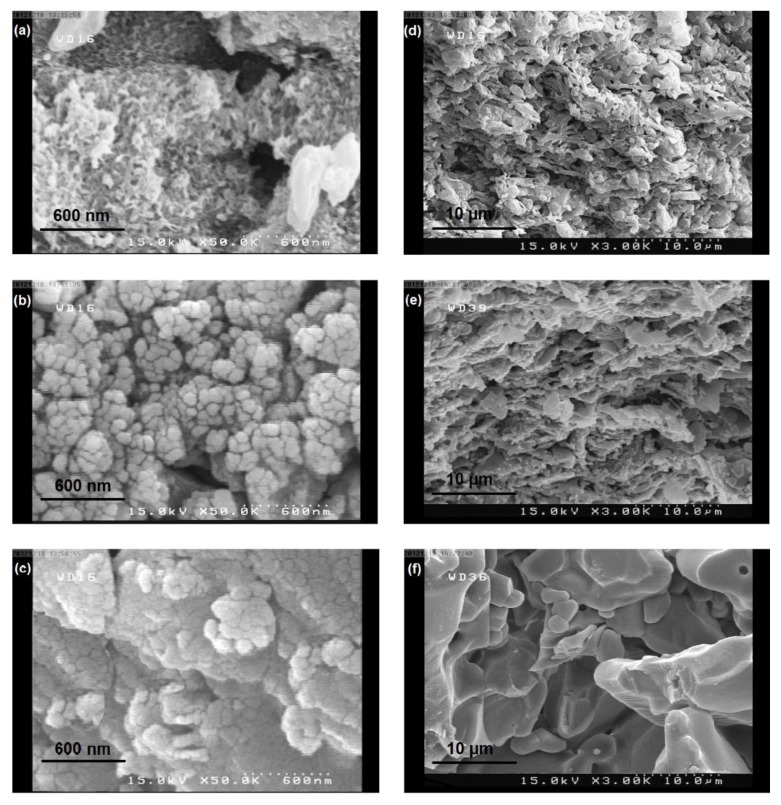
The FE-SEM images of the surface of supports: (**a**) mullite; (**b**) mullite-alumina; (**c**) α-alumina, cross-section of supports: (**d**) mullite; (**e**) mullite-alumina; (**f**) α-alumina.

Figure 3 shows surface FE-SEM images of ceramic supports used in the hybrid membranes. Both macropores and mesopores can be seen in mullite and mullite-alumina support surfaces (Figure 3a,b). However, the mullite-alumina support surface is more uniform than the mullite one and fewer macropores are seen there. As can be seen in Figure 3c, the outer surface of the commercial α-alumina support is more uniform and is almost free of macropores. Therefore, it can be concluded that as a result of the uniformity of the α-alumina support surface, membranes laid on this support have a uniform thickness all over the support surface. In contrast, for the synthesized supports and especially the mullite support, the polymer solution penetrates into the macropores and reduces the uniformity of the polymer layer thickness on the support. This means that in the macroporous regions, a higher amount of polymer solution penetrates and increases the thickness of the membrane. This phenomenon is more pronounced for solutions with lower viscosity. The non-uniformity of the polymer membrane layer on the ceramic support leads to a reduction in permeation flux and membrane selectivity. Figure 3d–f illustrate the cross-section of ceramic supports. It is clear that the structure of supports synthesized in this work is denser than the commercial ones and this increases the flow resistance for the permeating molecules.

### 3.2. Membrane Characterization

**Figure 4:**
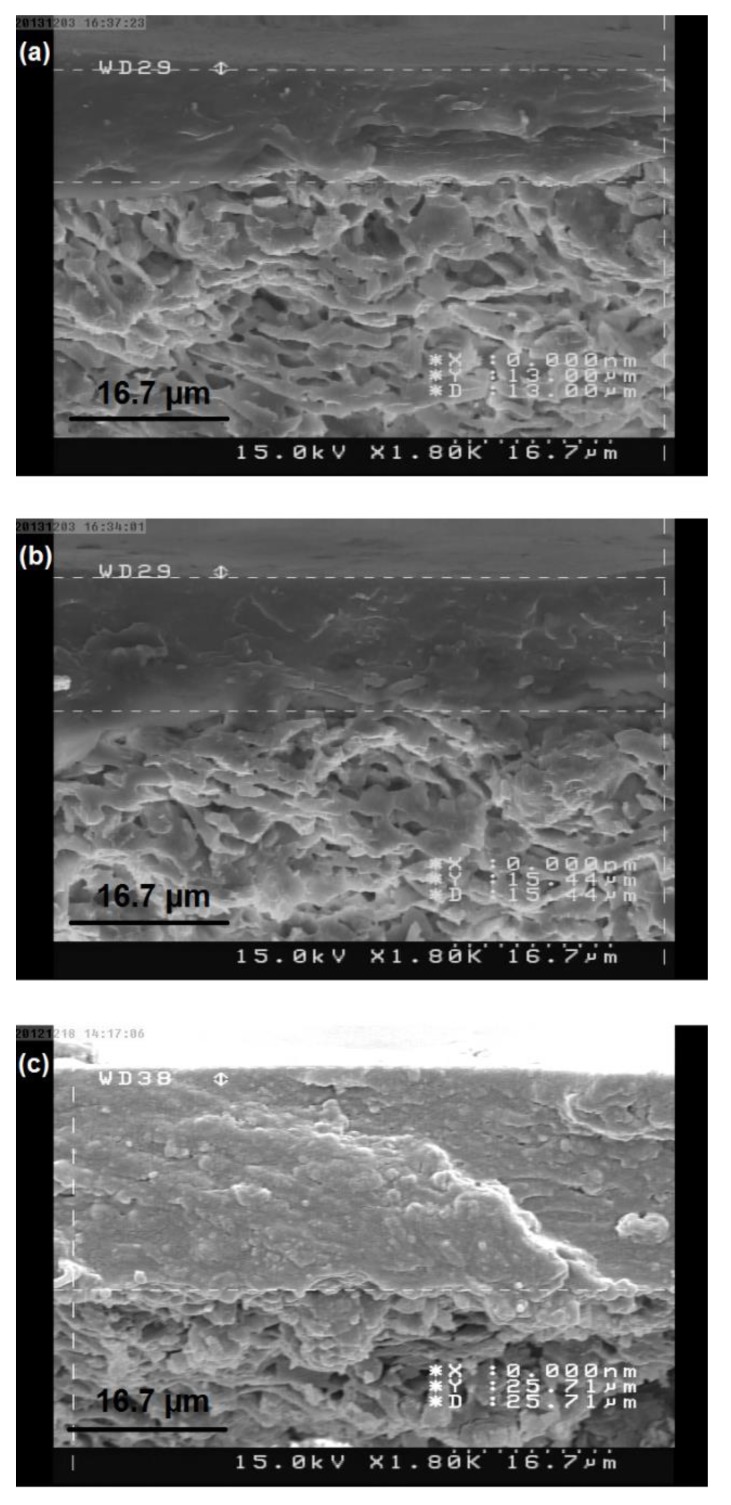
The FE-SEM images of the chitosan membrane cross section at different concentrations. (**a**) 2 wt.%; (**b**) 3 wt.%; (**c**) 4 wt.%.

Increasing the polymer concentration results in a decrease in the amount of the solvent. After drying the membrane, a small fraction of the mentioned layer will be removed. In other words, by increasing the polymer concentration, the layer thickness will decrease less during the drying process. Therefore, the polymer layer thickness will increase and this will naturally increase the flow resistance and decrease permeability. This hypothesis is confirmed by Figure 4 illustrating the membranes cross sections at different concentrations. The mean polymer layer thickness of the membranes with chitosan concentrations of 2%, 3%, and 4% is 13, 15.4, and 25.7 μm, respectively. Pore penetration in mullite support macropores can be seen in the membrane with 2% chitosan concentration (Figure 4a). Figure 4b,c show that polymer penetration decreases in mullite support macropores with 3 and 4 percent concentrations as a result of an increased solution viscosity, and the membrane thickness will thus be more uniform.

### 3.3. Pervaporation Experiments

In this research, the dehydration of ethanol in a pervaporation process has been studied in order to evaluate the hybrid membranes’ performance. Table 3 lists the obtained results of 9 synthesized membranes in the pervaporative ethanol dehydration at a temperature of 60 °C and a 90% concentration of ethanol in the feed. The effect of operational conditions on membrane performance will be discussed next.

#### 3.3.1. The Effect of Ceramic Supports on the Performance of Membranes

**Figure 5:**
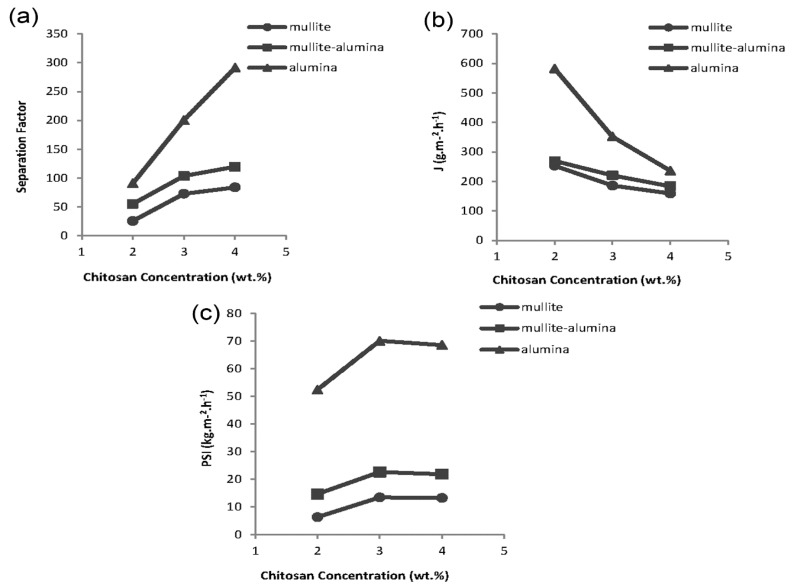
The pervaporation performance of membranes with different chitosan concentrations covered on different supports. (**a**) separation factor; (**b**) permeation flux; (**c**) pervaporation separation index (*PSI*).

Using a ceramic support increases the mechanical and thermal strengths as well as the processability of the hybrid membranes. Furthermore, ceramic supports might indirectly influence the permeation and selectivity of membranes in a pervaporation process. As previously discussed on the contact angle results (Figure 2b), α-alumina supports have the highest hydrophilicity and are more compatible with the hydrophilic chitosan polymer. Figure 5 shows the changes in the separation factor and permeation flux for ceramic supports at different chitosan concentrations. The membranes covered on mullite supports have the lowest separation factors while those on commercial α-alumina supports have the highest. According to the FE-SEM images in Figure 3, the surface of mullite and mullite-alumina supports have macropores that are not as uniform as α-alumina supports. Therefore, the polymer solution penetrates into these pores leading to an increased polymer layer thickness in those spots. Therefore, the membrane layer will not be uniform after drying. Under these conditions, the liquid molecules tend to pass through thin regions having less resistance against diffusion. The lower thickness of membranes as will be discussed in the next section results in a decrease in the water separation factor.

The permeation flux for chitosan membranes covered on the combined mullite-alumina support is higher than the mullite support and the maximum values were obtained for membranes covered on the commercial α-alumina supports. Due to the lower compaction and higher porosity of the α-alumina support cross-section (Figure 3), the permeated molecules have a better chance to pass through the membranes covered on these supports. Moreover, in the case of membranes covered on mullite supports, due to the non-uniformity of the thickness of the coated layer, the thicker section of the membranes is practically non-existent. Therefore, a less effective surface is exposed to the feed, resulting in a decrease in membrane permeate flux. This phenomenon is more pronounced in the case of membranes made by a lower chitosan concentration in which the lower viscosity of the chitosan solution facilitates the polymer penetration into the surface macropores. Hence, in the case of membranes made by 2% chitosan, the difference between the permeability of membranes covered on commercial support and the others is more considerable.

#### 3.3.2. The Effect of Chitosan Solution Concentration on Membrane Performance

The membranes were synthesized at three concentrations of 2, 3, and 4 wt.% in order to study how the membranes perform with respect to the changes in chitosan solution concentration. As mentioned in Figure 4, the more concentrated chitosan solution is the thicker membrane layer will form. In the pervaporation process, a thicker polymer layer is more appropriate for a higher selectivity, but on the other hand, it lowers the membrane permeability. This tradeoff between selectivity and permeability is confirmed by Figure 5 showing the trend of the permeation flux and separation factor for selected membranes covered on different supports. Such a behavior is due to the separation mechanism for dense polymer membranes, which is solution-diffusion. Feed components, water, and ethanol are dissolved in the chitosan layer. However, because of the higher affinity of chitosan for water, it is dissolved more than in ethanol. Due to their concentration gradients across the membrane, permeants diffuse through the polymer from the feed side to the permeate side. The thicker and more compact membrane layer increases both the components’ diffusion resistances and also their diffusion difference [37]. Therefore, a rise in diffusion resistance leads to a decrease in permeability, while the increase in the diffusion rate difference causes an increase in selectivity.

In order to find the optimum concentration, the quantity previously defined as *PSI* must be used. Figure 5c shows the *PSI* changes versus the chitosan concentration in membranes covered on different supports; the membranes have the best pervaporation overall performance at a chitosan concentration of 3 wt.%.

#### 3.3.3. The Effect of Feed Temperature on Membrane Performance

To investigate the effect of the operational conditions of feed temperature and concentration on the pervaporation process, the S6 sample, having shown the best performance, was used.

As expected, the temperature change has a significant effect on membrane performance. By increasing the temperature of the feed which is in direct contact with the membrane surface, the polymer chain mobility increases and, consequently, the permeability will increase [38]. Furthermore, one can mention, the effect of the increase in feed vapor pressure, which can provide an extra driving force for transfer while the vacuum pressure inside the membrane is fixed. In the pervaporation process, the feed enters in the liquid phase and the permeate exits in the vapor phase. It can be concluded that the liquid will turn into vapor somewhere in the middle of the membrane. By increasing the temperature, the phase transfer from liquid to vapor pushes the feed and the permeated molecule to pass through a larger part of the membrane thickness in the vapor phase. Since the velocity in the vapor phase is greater than that in the liquid phase, one observes an increase in permeability.

**Figure 6:**
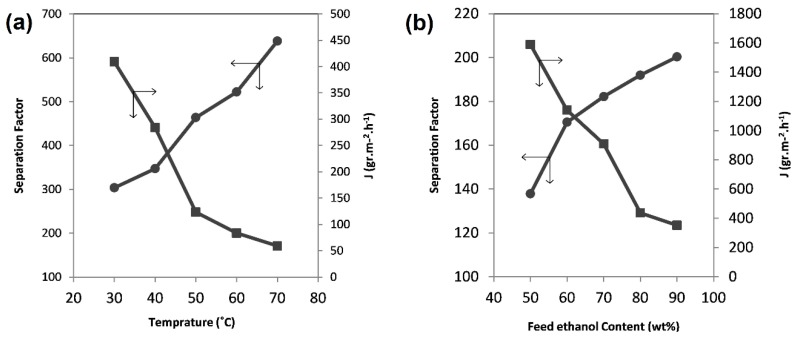
The pervaporation performance of the S6 membrane in different (**a**) temperatures; (**b**) feed ethanol content.

The mobility of the polymer chain increases by an increase in temperature and more space is created (membrane swelling), so ethanol molecules can pass through the membrane. Therefore, at higher temperatures, ethanol has a better chance to pass through the membrane and the selectivity decreases. Figure 6a depicts the change in selectivity and permeability as a function of temperature for the S6 sample.

#### 3.3.4. The Effect of Feed Concentration on Membrane Performance

The dependence of the pervaporation process on feed concentration was investigated in the range of the ethanol weight fraction of 50–90% and at a feed temperature of 60 °C. A reduction in ethanol fraction (which is equivalent to an increase in water fraction) in the feed for the S6 sample, as shown in Figure 6b, results in an increase in permeability and a decrease in selectivity for the membranes. As mentioned before, the swelling of the polymer chains in the water solution is one of the disadvantages of polymer membranes such as chitosan. Therefore, by increasing the water weight fraction in the feed, the swelling of chitosan chains may increase, resulting in a less-selective passage of feed through the membrane. Thus, the membrane permeability increases while its selectivity decreases.

## 4. Conclusions

Mullite and mullite-alumina tubular supports were fabricated from kaolin clay and alumina powder and successfully coated by the polymeric chitosan layer. The hydrophilicity and pore uniformity of ceramic supports affect the pervaporation performance of the hybrid membranes. The best pervaporation performance belonged to the membranes coated on commercial α-alumina supports due to uniform macropores and more hydrophilicity. Increasing the chitosan concentration increased the membrane layer thickness, which led to a higher separation factor and lower permeation flux. The pervaporation tests showed an optimum of 3 wt.% for chitosan concentration. The permeation flux increased remarkably with increasing either water content or the feed temperature, whereas the separation factor decreased. However, a comparison between the fabricated membranes in this study and the reported chitosan membranes (Table 4) modified by various methods demonstrates that the ceramic-supported chitosan hybrid membranes are suitable candidates for the pervaporation dehydration of ethanol providing high mechanical resistance in aggressive operating conditions. The use of ceramic supports can also be combined with other modification techniques such as cross-linking or using fillers for obtaining chitosan membranes with a superior performance in pervaporative alcohol purification.

## Figures and Tables

**Table 1:** The analysis of the Kaolin clay.

Components	Percentage	Phases	Percentage	Mesh	Percentage
SiO_2_	61–62	Kaolinite	64.00	<32 μ	100
TiO_2_	0.40	Calcite	2.40	<20 μ	99
Al_2_O_3_	24–25	Quartz	27.00	<2 μ	45
Fe_2_O_3_	0.45–0.65	Feld spar	6.60		
K_2_O	0.40	Total	100		
Na_2_O	0.50				
L.O.I	9.5–10				
Total	100				

**Table 2:** The analysis of the α-Al_2_O_3_ powder.

Components	Percentage	Properties	Value
Al_2_O_3_	99.6	Specific Gravity	3.9
Na_2_O	0.35	Average Particle Size (µm)	1
SiO_2_	0.03	Specific Area (m^2^/g)	6
Fe_2_O_3_	0.03	Bulk Density (g/cc)	0.82
TiO_2_	0.006		
L.O.I	0.07		
Total	100		

**Table 3:** The results of ethanol dehydration pervaporation process at a temperature of 60 °C and a 90% concentration of ethanol in the feed.

Sample	Ceramic Support	Chitosan Concentration (wt.%)	Permeation Flux (g·m^−2^·h^−1^)	Separation Factor	*PSI* (kg·m^−2^·h^−1^)
S1	mullite	2	253.0	25.6	6.2
S2	mullite-alumina	2	269.3	55.3	14.6
S3	α-alumina	2	582.1	91	52.4
S4	mullite	3	186.1	72.8	13.4
S5	mullite-alumina	3	220.4	103.5	22.6
S6	α-alumina	3	351.7	200.3	70.1
S7	mullite	4	159.2	83.8	13.2
S8	mullite-alumina	4	183.7	119.6	21.8
S9	α-alumina	4	236.5	291	68.6

**Table 4:** The comparison between synthesized membranes in this study with the literature.

Membrane	Filler	Support	Feed	Temperature (°C)	Separation Factor	Permeate Flux (g·m^−2^·h^−1^)	Reference
Cs	-	α-alumina	Eth (90%)/W	60	200.3	351.7	This study
Cs	-	mullite-alumina	Eth (90%)/W	60	119.6	183.7	This study
Cs—Alginate	-	PES	Eth (95%)/W	30	85	-	[39]
Cs—PSS	-	α-Al_2_O_3_	Eth (90%)/W	70	904	495	[40]
Cs	-	BC	Eth (95%)/W	24	9.2	42,800	[41]
Cs—PVA	-	PAN	Eth (95%)/W	30	93.7	320	[42]
Cs	-	PAN	Eth (90%)/W	70	256	1247	[43]
Cs	-	ZrO_2_-Al_2_O_3_	Eth (90%)/W	60	3780	100	[31]
Cs—H_3_PO_4_	-		Eth (96%)/W	24	213	580	[22]
Cs—GA-MA	-	-	Eth (90%)/W	50	634	300	[44]
Cs	HY Zeolite (20%)	-	Eth (90%)/W	-	102	353	[12]
Cs	H-ZSM-5 Zeolite (8%)	-	Eth (90%)/W	25	29.4	56	[25]
Cs	TEOS	-	Eth (90%)/W	80	460	284	[45]
Cs	TiO_2_	-	Eth (90%)/W	80	196	340	[28]
Cs	CNT	-	Eth (90%)/W	40	574	293	[26]
PVA	Silica fume	Mullite	Eth (90%)/W	45	80	1200	[43]

Cs: Chitosan; PES: Polyethersolfune; PSS: poly(4-styrenesulfonic acid); BC: bacterial cellulose; PVA: poly (vinyl alcohol); PAN: polyacrylonitrile; GA: glutaraldehyde; MA: maleic acid; CNT: carbon nanotubes.
